# Virome Diversity and Zoonotic Risks of *Haemaphysalis concinna* Ticks in Northeastern China

**DOI:** 10.1155/tbed/2710973

**Published:** 2026-09-25

**Authors:** Jiannan Liu, Qiqi Guo, Jixu Li, Zheng Gui, Ziyan Liu, Yuanning Ren, Yu Liu, Jingge Ma, Ning Liu, Jingfeng Yu, Lichao Sun, Zedong Wang

**Affiliations:** ^1^ Department of Infectious Diseases, Center of Infectious Diseases and Pathogen Biology, State Key Laboratory of Zoonotic Diseases, The First Hospital of Jilin University, Changchun, China, jlu.edu.cn; ^2^ School of Basic Medicine, Inner Mongolia Medical University, Hohhot, China, immu.edu.cn; ^3^ Yanbian Center for Disease Control and Prevention, Yanji, China; ^4^ Department of Emergency Medicine, The First Hospital of Jilin University, Changchun, China, jlu.edu.cn

**Keywords:** *Haemaphysalis concinna*, metatranscriptomics sequencing, Northeastern China, ticks, virome

## Abstract

*Haemaphysalis concinna* is a prevalent tick vector in Northeastern China, exhibiting a broad host range and the capacity to transmit over 40 human pathogens. Although numerous virome studies have investigated this tick species in Northeastern China, individual‐level viral prevalence, coinfection dynamics, and the host spectra of the harbored pathogenic viruses remain poorly characterized. In this study, we conducted metatranscriptomic sequencing combined with TaqMan‐based real‐time quantitative PCR (RT‐qPCR) validation on individual ticks to systematically profile the RNA virome of 112 adult *H. concinna* collected across Northeastern China. A total of 13 RNA viruses spanning six families and one unclassified group were identified, including four confirmed human pathogens, Tick‐borne encephalitis virus (TBEV), Dabieshan tick virus (DBSTV), Songling virus (SGLV), and Wetland virus (WELV), alongside one potential human pathogen, Ji’an nairovirus (JANV). Individual‐tick RT‐qPCR assays revealed an overall viral positivity rate of 46.4%, with prevalence for specific viruses ranging from 0.9% to 13.4%. Notably, multiviral coinfections were detected in 10.7% of ticks (8.9% harboring two viruses and 1.8% harboring three), predominantly involving nairoviruses and rhabdoviruses. Phylogenetic analyses further confirmed that local viral strains clustered closely with endemic pathogenic lineages. Furthermore, literature‐based host spectrum analysis demonstrated that these pathogenic viruses possess extensive vertebrate host ranges. Our findings highlight extensive viral diversity, high infection rates, and frequent coinfections in *H. concinna*, underscoring its pivotal role in maintaining and transmitting emerging tick‐borne viruses and emphasizing the heightened risk of zoonotic spillover in Northeastern China.

## 1. Introduction

As a crucial vector distributed across Eurasia, *Haemaphysalis concinna* exhibits a broad host range (up to 119 vertebrate species) and carries over 40 human pathogens [1]. In China, *H. concinna* has been recorded in at least 21 provinces, with its principal focus in the northeast [1]. Northeastern China serves as an epidemiological hotspot for tick‐borne viral diseases, where more than 10 distinct pathogenic viruses have been identified [2]. Importantly, emerging tick‐borne viruses, including Songling virus (SGLV), Wetland virus (WELV), Xuecheng virus, and Dabieshan tick virus (DBSTV), exhibit high infection rates in local *H. concinna* populations [3–7].

Northeastern China possesses the country’s most abundant forest resources, primarily concentrated in the Daxing’an Mountains (DXAMs), Xiaoxing’an Mountains (XXAMs), and Changbai Mountains (CBMs) [8]. These vast, forested areas provide ideal habitats that support abundant tick populations. While numerous viral studies on prevalent tick species in Northeastern China have revealed remarkable RNA virus diversity [8–11], no study to date has specifically focused on *H. concinna* to systematically investigate individual‐level viral positivity rates and multivirus coinfection features.

Here, we used metatranscriptomic sequencing to systematically profile the RNA virome of *H. concinna* ticks across Northeastern China, followed by RT‐qPCR validation of target viruses in individual ticks. Our findings reveal viral diversity and complex coinfection profiles in *H. concinna*, highlighting the potential zoonotic and spillover risks posed by pathogenic viruses. These findings provide fundamental insights into microbial cooccurrence and ecological interactions at the individual‐tick level.

## 2. Materials and Methods

### 2.1. Sample Collection and Pretreatment

From April to July 2024, the questing ticks were collected by the flagging method in DXAM and CBM of Northeastern China. *H. concinna* ticks were identified using integrated morphological and molecular methods, as detailed in prior studies [12, 13]. Each tick was individually homogenized in 200 μL of Dulbecco’s modified eagle’s medium (DMEM) with stainless steel beads using a tissue homogenizer [8]. The lysates were centrifuged at 12,000 ×*g* for 10 min at 4°C. Subsequently, 20 μL of the supernatant from each sample was pooled for library construction.

### 2.2. Library Construction and Metatranscriptomic Sequencing

Following treatment with micrococcal nuclease (NEB, USA) at 37°C for 2 h, viral RNA was extracted from the pooled supernatants using the TIANamp Virus RNA Kit (TIANGEN, China). The sequencing library was prepared using the NEBNext Ultra RNA Library Prep Kit (NEB, USA) according to the manufacturer’s protocol, followed by 10 cycles of PCR amplification after adapter ligation. Finally, sequencing was performed on an Illumina NovaSeq 6000 platform by Tanpu Biological Technology (Shanghai, China). The abundance distribution of viral species in the studied samples is shown in Figure S1.

### 2.3. Raw Data Analysis and Virus Classification

Raw sequencing data were analyzed as described previously [8]. Briefly, following adapter trimming and quality filtering, raw reads were depleted of ribosomal RNA, host, and bacterial sequences using BBMap. Contigs were assembled using SPAdes v3.14.1 and SOAPdenovo v2.04 [14, 15]. Viral contigs were identified by BLAST + v2.10.0 searches against GenBank’s nt and nr databases and further filtered to remove host‐ and bacteria‐derived sequences. Finally, the identified viruses were classified according to the latest International Committee on Taxonomy of Viruses (ICTV) taxonomy guidelines.

### 2.4. Virus RNA Extraction and RT‐qPCR Detection

Viral RNA from tick samples was extracted using the MagaBio plus Virus DNA/RNA Purification Kit Ⅲ (BioFlux, China) and subsequently reverse‐transcribed into complementary DNA (cDNA) using the PrimeScript RT Master Mix (TaKaRa, Japan) according to the manufacturer’s instructions. To further verify the identified viral species, RT‐qPCR was performed using either primers and probes designed from the assembled viral contigs or those described in previous studies (Table S1). RT‐qPCR amplification was performed using the Premix Ex Taq (Probe qPCR) Kit (TaKaRa, Japan) under the following thermal cycling conditions: initial denaturation at 95°C for 30 s, followed by 50 cycles of denaturation at 95°C for 5 s and annealing/extension at 60°C for 30 s. Viral copy numbers were quantified using standard curve equations from the established RT‐qPCR assays (Figure S2), with the exception of tick‐borne encephalitis virus (TBEV) and SGLV, which were calculated using previously reported equations [16].

### 2.5. Phylogenetic and Sequence Identity Analyses

Representative reference viral sequences were retrieved from the GenBank database (Table S2) and aligned with sequences obtained in this study (Table S3) using ClustalW implemented in MEGA 7.0. Phylogenetic analyses were performed on the aligned datasets using the maximum‐likelihood method in MEGA 7.0, with the best‐fitting substitution model selected for each alignment according to standard model‐selection criteria [17]. Branch support was assessed via bootstrap analysis with 1,000 replicates, and bootstrap values ≥ 70% were displayed on the trees. Sequence identities among aligned sequences were calculated using MegAlign (DNAstar v7.0).

### 2.6. Host Spectrum of Identified Pathogenic Viruses

To delineate the host infection spectrum of the identified pathogenic and putative pathogenic tick‐borne viruses, we systematically searched NCBI Virus (https://www.ncbi.nlm.nih.gov/labs/virus/vssi/#/find-data/virus), Google Scholar, and PubMed using the following keywords: “Songling virus,” “Wetland virus,” “Dadong virus,” “Ji’an nairovirus,” “Yanbian Nairo tick virus 2,” “Antu virus,” “Dabieshan tick virus,” and “Tick‐borne encephalitis virus.” Among these viruses, WELV has also been reported as Dadong virus, and Ji’an nairovirus (JANV) has also been reported as the Yanbian Nairo virus 2 or Antu virus [4, 8, 9, 18, 19]. Host species associated with each virus were extracted from epidemiological and virological studies. Host‐virus associations for TBEV and DBSTV were visualized as interaction network diagrams using Flourish (https://flourish.studio). Similarly, the associations for SGLV, WELV, and JANV were mapped as a Sankey diagram using the Wei Sheng Xin platform (https://www.bioinformatics.com.cn).

## 3. Results

### 3.1. Tick Collection and Metatranscriptomic Sequencing

**Figure 1:**
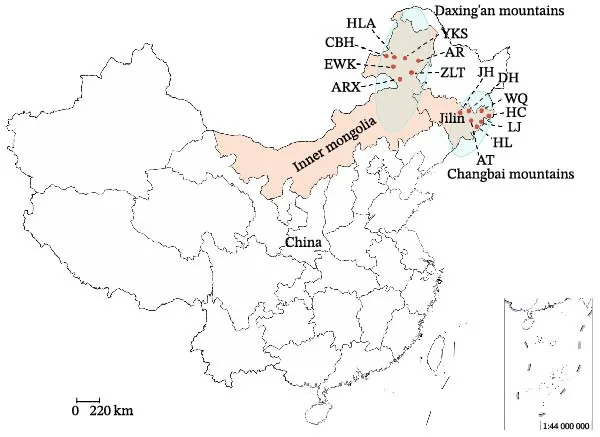
Sampling sites of *Haemaphysalis concinna* ticks in Northeastern China. Red dots indicate the collection locations, while the two major natural foci of tick‐borne diseases are highlighted with semitransparent light blue ovals. Abbreviations: AR, Arun; ARX, Arxan; AT, Antu; CBH, Chenbarhu; DH, Dunhua; EWK, Ewenki; HC, Hunchun; HL, Helong; HLA, Hailar; JH, Jiaohe; LJ, Longjing; WQ, Wangqing; YKS, Yakeshi; ZLT, Zhalantun.

A total of 112 adult *H. concinna* ticks, including 48 from CBM and 64 from DXAM, were sampled across 14 prefecture‐level cities and counties in Northeastern China (Figure 1 and Table S4). An RNA library was constructed and subjected to sequencing, yielding 4.1 GB of clean data and ~17.6 million nonribosomal RNA (non‐rRNA) reads. De novo assembly of ~8.7 million viral reads, which constituted 49.7% of the total non‐rRNA reads, generated a total of 42,347 viral contigs.

### 3.2. Virome of *H. concinna* Ticks

A total of 13 tick‐associated viruses were identified via sequencing. These viruses were taxonomically assigned to six established viral families: *Flaviviridae*, *Nairoviridae*, *Phenuiviridae*, *Rhabdoviridae*, *Tombusviridae*, and *Totiviridae*, as well as one unclassified viral species (Figure S1 and Table 1). All assembled viral sequences shared high nucleotide sequence identity with previously characterized species, yielding no novel viral species in this study. Specifically, these included TBEV (family *Flaviviridae*); JANV, SGLV, and WELV in family *Nairoviridae*; DBSTV (family *Phenuiviridae*); Tahe rhabdovirus 1 (THRV1), Yanbian rhabd tick 2 (YBRV2), and Yanbian rhabd tick 3 (YBRV3) in family *Rhabdoviridae*; Ningxia luteovirus (NXLV, family *Tombusviridae*); Daqing totiv tick virus 1 (DQTV1), Toti‐like virus 4 (TLV4), and Yanbian totiv tick virus 1 (YBTV1) in family *Orthototiviridae*, and Yanbian reovi tick virus 4 (YBRTV4) (Table 1).

**Table 1:** Viruses identified in the present study.

Classification	Virus (abbreviation)	Obtained sequence (bp)	Closest relative strain (% nt identity)
*Flaviviridae*			
* Orthoflavivirus*	Tick‐borne encephalitis virus (TBEV)	3427	Tick‐borne encephalitis virus NE‐SL4 (97.40)
*Nairoviridae*			
* Orthonairovirus*	Ji’an nairovirus (JANV)	L: 12,118	Ji’an nairovirus NE‐SDG2 (99.37)
* Orthonairovirus*	Ji’an nairovirus (JANV)	M: 4468	Ji’an nairovirus NE‐SDG2 (99.28)
* Orthonairovirus*	Ji’an nairovirus (JANV)	S: 1860	Ji’an nairovirus NE‐SDG2 (99.06)
* Orthonairovirus*	Songling virus (SGLV)	L: 12,079	Songling virus NE‐MEDG2 (98.65)
* Orthonairovirus*	Songling virus (SGLV)	M: 5663	Songling virus NE‐MEDG2 (97.97)
* Orthonairovirus*	Songling virus (SGLV)	S: 2158	Songling virus NE‐MEDG2 (97.19)
* Orthonairovirus*	Wetland virus (WELV)	L: 12,111	Wetland virus T3G2 (99.78)
* Orthonairovirus*	Wetland virus (WELV)	M: 4323	Wetland virus T3G2 (99.81)
* Orthonairovirus*	Wetland virus (WELV)	S: 1774	Wetland virus T3G2 (99.82)
*Phenuiviridae*			
* Uukuvirus*	Dabieshan tick virus (DBSTV)	L: 6196	Dabieshan tick virus HCCJ20 (98.80)
* Uukuvirus*	Dabieshan tick virus (DBSTV)	S: 752	Dabieshan tick virus HCCJ20 (99.20)
*Rhabdoviridae*			
* Alpharicinrhavirus*	Tahe rhabdovirus 1 (THRV1)	11,372	Tahe rhabdovirus 1 NE‐SL1 (99.59)
* Betaricinrhavirus*	Yanbian rhabd tick virus 2 (YBRV2)	11,047	Yanbian rhabd tick virus 2 TIGMIC 3 (99.62)
* Betaricinrhavirus*	Yanbian rhabd tick virus 3 (YBRV3)	11,149	Yanbian rhabd tick virus 3 TIGMIC 1 (98.86)
*Tombusviridae*			
* Luteovirus*	Ningxia luteovirus (NXLV)	2652	Ningxia luteovirus China‐NX143 (98.19)
*Orthototiviridae*			
	Daqing totiv tick virus 1 (DQTV1)	4930	Daqing totiv tick virus 1 TIGMIC_3 (95.41)
	Toti‐like virus 4 (TLV4)	4962	Toti‐like virus 4 China‐NX145 (95.42)
	Yanbian totiv tick virus 1 (YBTV1)	6280	Yanbian totiv tick virus 1 TIGMIC_74 (95.96)
Unclassified			
	Yanbian reovi tick virus 4 (YBRTV4)	2621	Yanbian reovi tick virus 4 TIGMIC 1 (99.22)

### 3.3. Viral Abundance, Prevalence, and Coinfection in *H. concinna* Ticks

**Figure 2:**
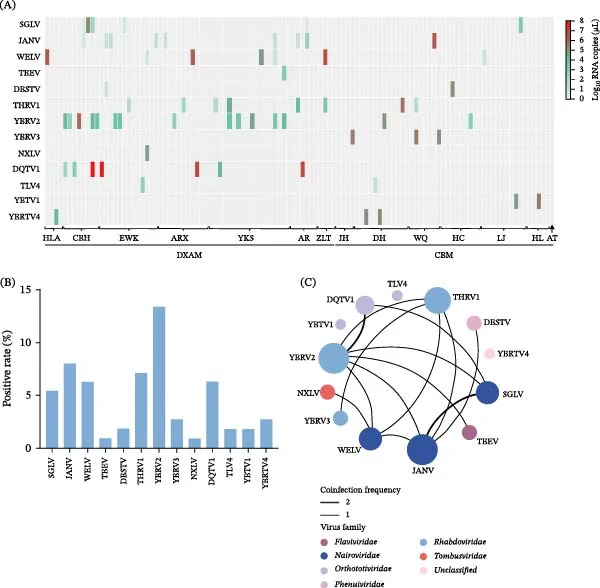
Viral abundance, prevalence, and coinfection in individual ticks. (A) Heatmap of viral abundance in individual ticks. (B) Viral prevalence across individual ticks. (C) Network graph illustrating viral coinfection events within individual ticks. Abbreviations: AR, Arun; ARX, Arxan; AT, Antu; CBH, Chenbarhu; DBSTV, Dabieshan tick virus; DH, Dunhua; DQTV1, Daqing totiv tick virus 1; EWK, Ewenki; HC, Hunchun; HL, Helong; HLA, Hailar; JANV, Ji’an nairovirus; JH, Jiaohe; LJ, Longjing; NXLV, Ningxia luteovirus; SGLV, Songling virus; TBEV, tick‐borne encephalitis virus; THRV1, Tahe rhabdovirus 1; TLV4, Toti‐like virus 4; WELV, Wetland virus; WQ, Wangqing; YBRTV4, Yanbian reovi tick virus 4; YBRV2, Yanbian rhabd tick virus 2; YBRV3, Yanbian rhabd tick virus 3; YBTV1, Yanbian totiv tick virus 1; YKS, Yakeshi; ZLT, Zhalantun.

All 13 target viruses were detected in individual‐tick samples using established RT‐qPCR assays. In total, eight viral species were identified in ticks collected from both DXAM and CBM, whereas three and two species were exclusively detected in ticks from DXAM and CBM, respectively (Figure 2A). Furthermore, for any given viral species, viral copy numbers exhibited substantial interindividual variation, with the highest viral loads primarily detected in ticks harboring DQTV1, WELV, and JANV. Tick samples with high viral copy numbers were used for genome amplification, yielding 14 nearly complete viral genomic sequences (Table S3).

Regarding prevalence, YBRV2 was the most frequently detected virus (13.4%), followed by JANV (8.0%), THRV1 (7.1%), WELV and DQTV1 (6.3% each), and SGLV (5.4%). Viruses with lower prevalence included YBRV3 and YBRTV4 (2.7% each), TLV4, YBTV1, and DBSTV (1.8% each), as well as NXLV and TBEV (0.9% each) (Figure 2B). Among the 112 collected ticks, 52 (46.4%) were positive for at least one viral species. Furthermore, 8.9% and 1.8% of the ticks harbored two and three viral species, respectively. Coinfection events predominantly involved nairoviruses and rhabdoviruses, both of which were shown to cooccur with other viral species within individual ticks (Figure 2C).

### 3.4. Homology and Phylogenetic Analysis of the Pathogenic Viruses

**Figure 3:**
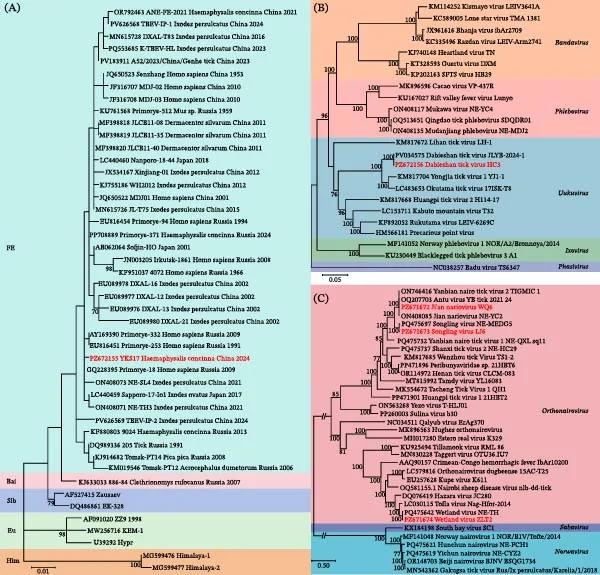
Phylogenetic analyses of the pathogenic viruses. Maximum likelihood trees were constructed based on the amino acid sequences of (A) the tick‐borne encephalitis virus E protein and the RNA‐dependent RNA polymerase (RdRp) of representative viruses within the (B) *Phenuiviridae* and (C) *Nairoviridae* families. Trees were generated in MEGA 7.0 using the JTT model. Bootstrap values >70 are displayed at the nodes. Strains sequenced in this study are highlighted in red.

Four of the viral species identified in this study have been confirmed to be pathogenic to humans, namely, TBEV, DBSTV, SGLV, and WELV [3, 4, 6, 20]. For TBEV, the identified strain shared 93.1%–97.6% nucleotide and 99.0%–99.6% amino acid identities in the E protein gene with known Far‐Eastern (FE) subtype strains from the DXAM region (Table S5). Phylogenetically, it clustered with endemic DXAM strains within the FE subtype clade (Figure 3A). For DBSTV, the obtained strain exhibited 98.2%–99.1% nucleotide and 99.2%–99.8% amino acid identities in the RNA‐dependent RNA‐polymerase (RdRp) gene relative to strains from Northeastern China (Table S6). RdRp‐based phylogeny placed this DBSTV strain within the *Uukuvirus* clade, alongside two other two‐segmented tick‐borne phenuiviruses, such as Yongjia tick virus 1 and Okutama tick virus (Figure 3B).

Among the three nairoviruses identified, WELV shared 99.8% nucleotide and 99.8%–99.9% amino acid identities in the RdRp gene with previously reported strains from Northeastern China. Similarly, SGLV exhibited 97.5%–98.7% nucleotide and 99.6%–99.7% amino acid identities with known strains (Table S7). Furthermore, although JANV has not been reported to infect humans, it shares 75%–78% nucleotide and 80%–85% amino acid identities with SGLV. Phylogenetically, WELV grouped robustly within the clade of canonical pathogenic nairoviruses, specifically, Crimean‐Congo hemorrhagic fever virus and the Nairobi sheep disease virus. Conversely, SGLV clustered within a distinct lineage of emerging tick‐borne nairoviruses, which includes JANV and human‐pathogenic viruses like Tacheng tick virus 1, Xuecheng virus, and Shanxi tick virus 2 (Figure 3C) [5, 6, 21].

### 3.5. Host Spectrum of Identified Pathogenic Viruses

**Figure 4:**
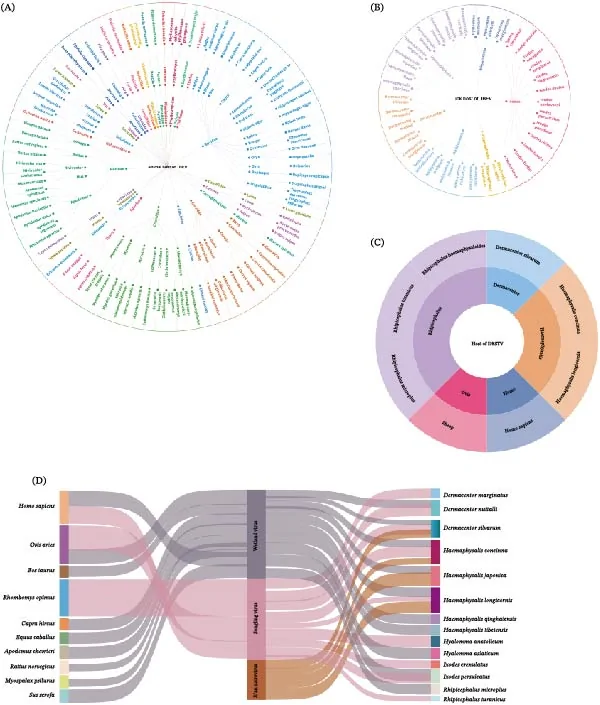
Host spectrum of the identified pathogenic viruses. (A) Vertebrate and (B) tick hosts of the tick‐borne encephalitis virus (TBEV). (C) Host spectrum of Dabieshan tick virus (DBSTV). (D) Host spectrum of Songling virus, Ji’an nairovirus, and Wetland virus.

Based on a systematic literature review, TBEV has been identified in at least 91 vertebrate host species and 36 tick species (Figure 4A,B). As for phenuiviruses, DBSTV has been detected in humans and sheep, as well as in six tick species (Figure 4C). Among the nairoviruses, WELV was reported in at least nine vertebrate and 10 tick species, and SGLV in three vertebrate and nine tick species, while JANV was detected exclusively in four tick species (Figure 4D).

## 4. Discussion

This study characterizes the virome of *H. concinna* ticks collected from two major natural foci of tick‐borne viruses in Northeastern China. The findings reveal extensive viral diversity and complex coinfection profiles in the ticks. Moreover, we identified four tick‐borne viruses known to infect humans, underscoring the public health significance of *H. concinna* as a potential vector.

The rapid development and wide application of high‐throughput sequencing technology have advanced tick‐borne pathogen surveillance in the past decade [8, 9, 22, 23]. Interspecies variations in tick habitat, geographic range, and host specificity lead to distinct differences in pathogen carriage and transmission, directly shaping prevention and control strategies for tick‐borne diseases [24]. Therefore, conducting virome studies on specific tick species within particular regions and analyzing viral coinfections in individual ticks are crucial for the comprehensive prevention and control of tick‐borne diseases. In this context, a targeted investigation of *H. concinna* is scientifically warranted, given its distinct ecological niche and established role as a bridge vector linking wildlife reservoirs to human populations [1].

Unlike previous tick virome studies [8, 10, 11], our approach involved grinding individual ticks prior to sample pooling, followed by RT‐qPCR verification. This strategy not only circumvents the high costs of individual high‐throughput sequencing but also minimizes cross‐contamination and boosts detection sensitivity. Ultimately, this methodological refinement revealed a high overall viral infection rate (46.4%) and demonstrated that 8.9% and 1.8% of ticks concurrently harbored two or three viral species, respectively (Figure 2). Crucially, these coinfections predominantly involved the rhabdoviruses and the three known or potentially pathogenic nairoviruses. Thus, while our virome analysis did not identify any novel viral species, it provides a fresh perspective on the viral cooccurrence landscape within individual *H. concinna* ticks.

It has been well documented that individual ticks frequently harbor both endosymbionts and multiple tick‐borne pathogens [25]. This cooccurrence of distinct pathogens can facilitate host infection through mutual enhancement [26–29]. Similarly, multiviral coinfections in ticks may facilitate the establishment of viral pathogens in vertebrate hosts via diverse mechanistic pathways. Nevertheless, empirical evidence remains scarce, underscoring the need for rigorous investigation into the underlying molecular and ecological mechanisms.

In the present study, we identified four known human‐pathogenic viruses. Of these viruses, TBEV is widely distributed in Northeastern China and is primarily identified in *Ixodes persulcatus* [20, 30]. Unlike TBEV, however, DBSTV, SGLV, and WELV were detected at much higher prevalence in *H. concinna* compared to other tick species [3, 4, 7]. Our analysis also indicates that all these pathogenic viruses share a broad range of vertebrate hosts (Figure 4). Taken together, the wide hosts, coupled with the feeding habits of *H. concinna* [1] not only highlights the zoonotic potential of these viruses but also suggests a risk of viral spillover to humans via alternative routes beyond direct tick bites (e.g., contact with infected animals or their products).

This study presents several limitations. First, the relatively small sample size of *H. concinna* constrained the detection of certain viruses, precluding a complete characterization of the viral coinfection landscape within this tick population in Northeastern China. Second, our analysis focused exclusively on viruses, omitting bacterial and protozoan pathogens. As a result, our profiling of pathogen coinfections in *H. concinna* remains partial, which may underestimate both the complexity of multipathogen interactions and the associated ecological or transmission dynamics. Nonetheless, as a preliminary investigation, our findings pave the way for future research exploring the cocarriage and complex interplay of diverse microorganisms at the individual‐tick level.

## 5. Conclusions

In summary, we identified 13 RNA viruses (across six families and one unclassified) in *H. concinna* ticks from Northeastern China. The ticks exhibited a high overall carriage rate (46.4%) and frequent coinfections, with 8.9% and 1.8% of ticks harboring two or three viral species, respectively. Notably, the detected viruses included four confirmed human pathogens and one potential human pathogen, with prevalence rates ranging from 0.9% to 8.0%. The broad host ranges of these viruses, together with that of *H. concinna*, collectively highlight their potential for zoonotic transmission and spillover.

## Author Contributions

**Jiannan Liu**: experimental work, data curation, formal analysis, investigation, methodology, software, visualization, writing – original draft. **Qiqi Guo**: experimental work, data curation, formal analysis, methodology, visualization, writing – review and editing. **Jixu Li, Zheng Gui, Ziyan Liu, Jingge Ma, Ning Liu, and Jingfeng Yu**: methodology, supervision, writing – review and editing. **Yuanning Ren**: experimental work, data curation. **Yu Liu**: sample collection, writing – review and editing. **Lichao Sun**: investigation, project administration, resources, data curation, formal analysis, methodology, visualization, writing – original draft, writing – review and editing, manuscript revision. **Zedong Wang**: funding acquisition, sample collection, investigation, project administration, resources, methodology, writing – original draft, writing – review and editing, manuscript revision.

## Funding

This study was supported by the National Natural Science Foundation of Jilin Province, China (Grant 20240101017JC).

## Disclosure

The funders had no role in study design, data collection and analysis, decision to publish, or preparation of the manuscript.

## Ethics Statement

The authors have nothing to report.

## Conflicts of Interest

The authors declare no conflicts of interest.

## Supporting Information

Additional supporting information can be found online in the Supporting Information section.

## Supporting information


**Supporting Information** Table S1: Primers and probes used in this study. Table S2: Reference viral sequences used in the study. Table S3: Identified viral strains in the present study. Table S4: Summary of tick sample collection. Table S5: Nucleotide (lower left) and amino acid (upper right) sequence identities of the TBEV E gene. Table S6: Nucleotide (lower left) and amino acid (upper right) sequence identities of the DBSTV RdRp gene. Table S7: Nucleotide (lower left) and amino acid (upper right) sequence identities of the SGLV, JANV, and WELV RdRp genes. Figure S1: Composition and relative abundance of viral families identified in the samples. Figure S2: Standard curves for the 11 tick‐borne viruses.

## Data Availability

The data that support the findings of this study are available in the Supporting Information. All sequence reads have been deposited in the Short Read Archive (BioProject ID: PRJNA1528486), and the viral genomes have been submitted to GenBank (Table S3).
