# Supplementary material for: Virome Diversity and Zoonotic Risks of Haemaphysalis concinna Ticks in Northeastern China
Source: Transbound Emerg Dis. 2026 Sep 25;2026:2710973. doi: 10.1155/tbed/2710973 (PMC13614070; doi:10.1155/tbed/2710973)
Supplement: Supplementary file 1 [file TBED-2026-2710973-s001.docx]

**Supplementary materials**

Table S1. Primers and probes used in this study.

| **Primers/probes** | **Sequence** |
| --- | --- |
| TBEV-F | 5’-CAYGCAAAGCTRTCGGATACCA-3’ |
| TBEV-R | 5’-YGGTGYTCTTCAGCCARRGT-3’ |
| TBEV-P | 5’-(FAM)-TYGCGGCCAGATGCCCHACAA-(BHQ1)-3’ |
| SGLV-F | 5’-AGATTGTGCATGCTGCRTC-3’ |
| SGLV-R | 5’-GTTTGTAAGATGCTTTCTTT-3’ |
| SGLV-P | 5’-(FAM)-YCAAYGATGATGGTTTGACCAACCAACA-(BHQ1)-3’ |
| JANV-F | 5’-AGGTCCTTRYTGGCRAAG-3’ |
| JANV-R | 5’-CCTTCAGTTCTHCCAGTC-3’ |
| JANV-P | 5’-(FAM)-ACGGACTTGACAGRACAGACCTT-(BHQ1)-3’ |
| WELV-F | 5’-CGAGGCCACTAGATTCTG-3’ |
| WELV-R | 5’-CCAGACCTTGACAATGTC-3’ |
| WELV-P | 5’-(FAM)-AGTGTGCTTGGTCCTCCTCTAC-(BHQ1)-3’ |
| DBSTV-F | 5’-CACGTTGAGAGTACCAAG-3’ |
| DBSTV-R | 5’-GTCAGGAGTCAGTAAGTC-3’ |
| DBSTV-P | 5’-(FAM)-AGCCAGACCTTCATTCACGACT-(BHQ1)-3’ |
| THRV1-F | 5’-CAGCAACGTGGTTTACTG-3’ |
| THRV1-R | 5’-GTGTGGTCCTGACTTGAG-3’ |
| THRV1-P | 5’-(FAM)-CCAGCAGACTAAGCCTCGGT-(BHQ1)-3’ |
| YBRV2-F | 5’-GGCTAAGGAAATCTTCTC-3’ |
| YBRV2-R | 5’-CCAGTGTTCTAGACTGTA-3’ |
| YBRV2-P | 5’-(FAM)-ATGAACTACTACCAGAAGAACCGACG-(BHQ1)-3’ |
| YBRV3-F | 5’-TCCCTTGCATTTGTGAAG-3’ |
| YBRV3-R | 5’-GGAAGATGTGAGAAGTGG-3’ |
| YBRV3-P | 5’-(FAM)-CTCAGGTCTCATCCAGTTCTCCG-(BHQ1)-3’ |
| NXLV-F | 5’-ACGTGTTGAGTACTACAA-3’ |
| NXLV-R | 5’-CGCCGATATCAAYCTATA-3’ |
| NXLV-P | 5’-(FAM)-TTGAGAACCGTATCCGCCAGT-(BHQ1)-3 |
| DQTV1-F | 5’-GTAGGTATGACATAACTACG-3’ |
| DQTV1-R | 5’-GTCTCTTATGAACCTCTTG-3’ |
| DQTV1-P | 5’-(FAM)-CACTAATGAAGCCACGCAGACAG-(BHQ1)-3’ |
| TLV4-F | 5’-GCTGAAAGGAGGTTCATA-3’ |
| TLV4-R | 5’-CAGTCCAAAGATGCTGTC-3’ |
| TLV4-P | 5’-(FAM)-CTTGGAAGCAGGTCTACTCMGA-(BHQ1)-3’ |
| YBTV1-F | 5’-GACTCMACYGTCAGAAGG-3’ |
| YBTV1-R | 5’-CGTAGAAYGAGATGGAKC-3’ |
| YBTV1-P | 5’-(FAM)-TCGTGAACGCWTCCGGYCTG-(BHQ1)-3’ |
| YBRTV4-F | 5’-CTTCCYCTGTGGAACGAG-3’ |
| YBRTV4-R | 5’-TYGCGRATCATRGTATCC-3’ |
| YBRTV4-P | 5’-(FAM)-CATCCTGACGCCTCCACTCT-(BHQ1)-3’ |

Table S2. Reference viral sequences used in the study.

| **Family** | | **Genus** | **Virus** | **Strain** | **GenBank accession No.** |
| --- | --- | --- | --- | --- | --- |
| ***Flaviviridae*** | | | | | |
|  | *Flaviviridae* | *Orthoflavivirus* | Tick-borne encephalitis virus | Sofjin-HO | AB062064 |
|  | *Flaviviridae* | *Orthoflavivirus* | Tick-borne encephalitis virus | Zausaev | AF527415 |
|  | *Flaviviridae* | *Orthoflavivirus* | Tick-borne encephalitis virus | K23 | AM600965 |
|  | *Flaviviridae* | *Orthoflavivirus* | Tick-borne encephalitis virus | Primorye-332 | AY169390 |
|  | *Flaviviridae* | *Orthoflavivirus* | Tick-borne encephalitis virus | EK-328 | DQ486861 |
|  | *Flaviviridae* | *Orthoflavivirus* | Tick-borne encephalitis virus | 205 | DQ989336 |
|  | *Flaviviridae* | *Orthoflavivirus* | Tick-borne encephalitis virus | 886-84 | EF469662 |
|  | *Flaviviridae* | *Orthoflavivirus* | Tick-borne encephalitis virus | DXAL-16 | EU089978 |
|  | *Flaviviridae* | *Orthoflavivirus* | Tick-borne encephalitis virus | DXAL-18 | EU089979 |
|  | *Flaviviridae* | *Orthoflavivirus* | Tick-borne encephalitis virus | DXAL-21 | EU089980 |
|  | *Flaviviridae* | *Orthoflavivirus* | Tick-borne encephalitis virus | Primorye-18 | GQ228395 |
|  | *Flaviviridae* | *Orthoflavivirus* | Tick-borne encephalitis virus | MDJ-03 | JF316708 |
|  | *Flaviviridae* | *Orthoflavivirus* | Tick-borne encephalitis virus | MDJ-02 | JF316707 |
|  | *Flaviviridae* | *Orthoflavivirus* | Tick-borne encephalitis virus | MDJ01 | JQ650522 |
|  | *Flaviviridae* | *Orthoflavivirus* | Tick-borne encephalitis virus | Senzhang | JQ650523 |
|  | *Flaviviridae* | *Orthoflavivirus* | Tick-borne encephalitis virus | Xinjiang-01 | JX534167 |
|  | *Flaviviridae* | *Orthoflavivirus* | Tick-borne encephalitis virus | Zabaikalye 30-00 | KC422667 |
|  | *Flaviviridae* | *Orthoflavivirus* | Tick-borne encephalitis virus | Zabaikalye 6-09 | KF826915 |
|  | *Flaviviridae* | *Orthoflavivirus* | Tick-borne encephalitis virus | 9024 | KF880803 |
|  | *Flaviviridae* | *Orthoflavivirus* | Tick-borne encephalitis virus | WH2012 | KJ755186 |
|  | *Flaviviridae* | *Orthoflavivirus* | Tick-borne encephalitis virus | Sib-XJ-X5 | KP345889 |
|  | *Flaviviridae* | *Orthoflavivirus* | Tick-borne encephalitis virus | Sapporo-17-Io1 | LC440459 |
|  | *Flaviviridae* | *Orthoflavivirus* | Tick-borne encephalitis virus | Nanporo-18-44 | LC440460 |
|  | *Flaviviridae* | *Orthoflavivirus* | Tick-borne encephalitis virus | JLCB11-08 | MF398818 |
|  | *Flaviviridae* | *Orthoflavivirus* | Tick-borne encephalitis virus | JLCB11-35 | MF398819 |
|  | *Flaviviridae* | *Orthoflavivirus* | Tick-borne encephalitis virus | JLCB11-40 | MF398820 |
|  | *Flaviviridae* | *Orthoflavivirus* | Tick-borne encephalitis virus | Himalaya-1 | MG599476 |
|  | *Flaviviridae* | *Orthoflavivirus* | Tick-borne encephalitis virus | Himalaya-2 | MG599477 |
|  | *Flaviviridae* | *Orthoflavivirus* | Tick-borne encephalitis virus | NE-TH3 | ON408071 |
|  | *Flaviviridae* | *Orthoflavivirus* | Tick-borne encephalitis virus | NE-SL4 | ON408073 |
|  | *Flaviviridae* | *Orthoflavivirus* | Tick-borne encephalitis virus | ANE-FE-2021 | OR792463 |
|  | *Flaviviridae* | *Orthoflavivirus* | Tick-borne encephalitis virus | Primorye-371 | PP708889 |
|  | *Flaviviridae* | *Orthoflavivirus* | Tick-borne encephalitis virus | MBC045 | PP862726 |
|  | *Flaviviridae* | *Orthoflavivirus* | Tick-borne encephalitis virus | K-TBEV-HL | PQ553685 |
|  | *Flaviviridae* | *Orthoflavivirus* | Tick-borne encephalitis virus | HLB-ZH148 | PQ790053 |
|  | *Flaviviridae* | *Orthoflavivirus* | Tick-borne encephalitis virus | HLB-H168 | PQ790054 |
|  | *Flaviviridae* | *Orthoflavivirus* | Tick-borne encephalitis virus | HLB-H372 | PQ790056 |
|  | *Flaviviridae* | *Orthoflavivirus* | Tick-borne encephalitis virus | A52/2023/China/Genhe | PV183911 |
|  | *Flaviviridae* | *Orthoflavivirus* | Tick-borne encephalitis virus | TBEV-IP-1 | PV626568 |
|  | *Flaviviridae* | *Orthoflavivirus* | Tick-borne encephalitis virus | TBEV-IP-2 | PV626569 |
|  | *Flaviviridae* | *Orthoflavivirus* | Tick-borne encephalitis virus | HLB-T74 | MN615727 |
|  | *Flaviviridae* | *Orthoflavivirus* | Tick-borne encephalitis virus | Hypr | U39292 |
| ***Phenuiviridae*** | | | | | |
|  | *Phenuiviridae* | *Bandavirus* | Kismayo virus | LEIV3641A | KM114252 |
|  | *Phenuiviridae* | *Bandavirus* | Lone star virus | TMA 1381 | KC589005 |
|  | *Phenuiviridae* | *Bandavirus* | Bhanja virus | ibAr2709 | JX961616 |
|  | *Phenuiviridae* | *Bandavirus* | Razdan virus | LEIV-Arm2741 | KC335496 |
|  | *Phenuiviridae* | *Bandavirus* | Heartland virus | TN | KJ740148 |
|  | *Phenuiviridae* | *Bandavirus* | Guertu virus | DXM | KT328593 |
|  | *Phenuiviridae* | *Bandavirus* | SFTS virus | HB29 | KP202163 |
|  | *Phenuiviridae* | *Phlebovirus* | Cacao virus | VP-437R | MK896596 |
|  | *Phenuiviridae* | *Phlebovirus* | Rift Valley fever virus | Lunyo | KU167027 |
|  | *Phenuiviridae* | *Phlebovirus* | Mukawa virus | NE-YC4 | ON408117 |
|  | *Phenuiviridae* | *Phlebovirus* | Qingdao tick phlebovirus | SDQDR01 | OQ513651 |
|  | *Phenuiviridae* | *Phlebovirus* | Mudanjiang phlebovirus | NE-MDJ2 | ON408135 |
|  | *Phenuiviridae* | *Uukuvirus* | Lihan Tick Virus | LH-1 | KM817672 |
|  | *Phenuiviridae* | *Uukuvirus* | Dabieshan tick virus | JLYB-2024-1 | PV034575 |
|  | *Phenuiviridae* | *Uukuvirus* | Dabieshan tick virus | TIGMIC 38 | ON812294 |
|  | *Phenuiviridae* | *Uukuvirus* | Dabieshan tick virus | HCSQ34 | PQ059386 |
|  | *Phenuiviridae* | *Uukuvirus* | Dabieshan tick virus | HCSQ69 | PQ059376 |
|  | *Phenuiviridae* | *Uukuvirus* | Dabieshan tick virus | HCCJ14 | PQ059392 |
|  | *Phenuiviridae* | *Uukuvirus* | Dabieshan tick virus | NE-TMXP5 | PV221372 |
|  | *Phenuiviridae* | *Uukuvirus* | Dabieshan tick virus | HCCJ20 | PQ059394 |
|  | *Phenuiviridae* | *Uukuvirus* | Dabieshan tick virus | HCSQ62 | PQ059374 |
|  | *Phenuiviridae* | *Uukuvirus* | Dabieshan tick virus | HCCJ2 | PQ059390 |
|  | *Phenuiviridae* | *Uukuvirus* | Dabieshan tick virus | TMSQ5 | PQ059362 |
|  | *Phenuiviridae* | *Uukuvirus* | Dabieshan tick virus | TMSQ8 | PQ059366 |
|  | *Phenuiviridae* | *Uukuvirus* | Dabieshan tick virus | NE-BXXP1 | PV221352 |
|  | *Phenuiviridae* | *Uukuvirus* | Dabieshan tick virus | tick/Shandong/SDLY14/2019 | OM368057 |
|  | *Phenuiviridae* | *Uukuvirus* | Yongjia tick virus 1 | YJ1-1 | KM817704 |
|  | *Phenuiviridae* | *Uukuvirus* | Okutama tick virus | 17ISK-T8 | LC483653 |
|  | *Phenuiviridae* | *Uukuvirus* | Huangpi tick virus 2 | H114-17 | KM817668 |
|  | *Phenuiviridae* | *Uukuvirus* | Kabuto mountain virus | T32 | LC153711 |
|  | *Phenuiviridae* | *Uukuvirus* | Rukutama virus | LEIV-6269C | KF892052 |
|  | *Phenuiviridae* | *Uukuvirus* | Precarious point virus | ~ | HM566181 |
|  | *Phenuiviridae* | *Ixovirus* | Norway phlebovirus 1 | NOR/A2/Bronnoya/2014 | MF141052 |
|  | *Phenuiviridae* | *Ixovirus* | Blacklegged tick phlebovirus 3 | A1 | KU230449 |
|  | *Phenuiviridae* | *Phasivirus* | Badu virus | TS6347 | NC_038257 |
| ***Nairoviridae*** | | | | | |
|  | *Nairoviridae* | *Orthonairovirus* | Yanbian Nairo tick virus 2 | TIGMIC 1 | ON746416 |
|  | *Nairoviridae* | *Orthonairovirus* | Antu virus | YB tick 2021 24 | OQ207703 |
|  | *Nairoviridae* | *Orthonairovirus* | Jian nariovirus | NE-YC2 | ON408085 |
|  | *Nairoviridae* | *Orthonairovirus* | Songling virus | NE-WEQH | PQ475681 |
|  | *Nairoviridae* | *Orthonairovirus* | Songling virus | NE-GH1 | PQ475680 |
|  | *Nairoviridae* | *Orthonairovirus* | Songling virus | NE-GPLC5 | PQ475686 |
|  | *Nairoviridae* | *Orthonairovirus* | Songling virus | NE-MEDG5 | PQ475697 |
|  | *Nairoviridae* | *Orthonairovirus* | Shanxi tick virus 2 | NE-HC29 | PQ475737 |
|  | *Nairoviridae* | *Orthonairovirus* | Yanbian Nairo tick virus 1 | NE-QXL sq1 | PQ475732 |
|  | *Nairoviridae* | *Orthonairovirus* | Peribunyaviridae sp. | 21HBT6 | PP471896 |
|  | *Nairoviridae* | *Orthonairovirus* | Henan tick virus | CLCM-083 | OR114972 |
|  | *Nairoviridae* | *Orthonairovirus* | Huangpi Tick Virus 1 | 21HBT2 | PP471901 |
|  | *Nairoviridae* | *Orthonairovirus* | Wenzhou Tick Virus | TS1-2 | KM817685 |
|  | *Nairoviridae* | *Orthonairovirus* | Tamdy virus | YL16083 | MT815992 |
|  | *Nairoviridae* | *Orthonairovirus* | Tacheng Tick Virus 1 | QH1 | MK554672 |
|  | *Nairoviridae* | *Orthonairovirus* | Yezo virus | T-HLJ01 | ON563268 |
|  | *Nairoviridae* | *Orthonairovirus* | Sulina virus | b30 | PP260003 |
|  | *Nairoviridae* | *Orthonairovirus* | Hughes orthonairovirus | Dry Tortugas | MK896563 |
|  | *Nairoviridae* | *Orthonairovirus* | Estero Real virus | K329 | MH017280 |
|  | *Nairoviridae* | *Orthonairovirus* | Qalyub virus | ErAg370 | NC_034511 |
|  | *Nairoviridae* | *Orthonairovirus* | Tillamook virus | RML 86 | KU925494 |
|  | *Nairoviridae* | *Orthonairovirus* | Taggert virus | OTU36.IU7 | MN830228 |
|  | *Nairoviridae* | *Orthonairovirus* | Crimean-Congo hemorrhagic fever virus | IbAr10200 | AAQ90157 |
|  | *Nairoviridae* | *Orthonairovirus* | Orthonairovirus dugbeense | 15AC-T25 | LC579816 |
|  | *Nairoviridae* | *Orthonairovirus* | Kupe virus | K611 | EU257628 |
|  | *Nairoviridae* | *Orthonairovirus* | Hazara virus | JC280 | DQ076419 |
|  | *Nairoviridae* | *Orthonairovirus* | Nairobi sheep disease virus | nlb-dd-tick | OQ581155 |
|  | *Nairoviridae* | *Orthonairovirus* | Tofla virus | Nag-Hfor-2014 | LC030115 |
|  | *Nairoviridae* | *Orthonairovirus* | Wetland virus | NE-TH | PQ475642 |
|  | *Nairoviridae* | *Orthonairovirus* | Wetland virus | T3G2 | OR860403 |
|  | *Nairoviridae* | *Orthonairovirus* | Wetland virus | PS1 | OR860402 |
|  | *Nairoviridae* | *Orthonairovirus* | Wetland virus | T8A1 | OR860405 |
|  | *Nairoviridae* | *Sabavirus* | South Bay virus | SC1 | KX184198 |
|  | *Nairoviridae* | *Norwavirus* | Beiji nairovirus | BJNV BSQG1734 | OR148703 |
|  | *Nairoviridae* | *Norwavirus* | Norway nairovirus 1 | NOR/B1V/Tofte/2014 | MF141048 |
|  | *Nairoviridae* | *Norwavirus* | Gakugsa tick virus | Rus/Ix persulcatus/Karelia/1/2018 | MN542362 |
|  | *Nairoviridae* | *Norwavirus* | Yichun nairovirus | NE-CYZ2 | PQ475619 |
|  | *Nairoviridae* | *Norwavirus* | Hunchun nairovirus | NE-FCH1 | PQ475621 |
| ***Rhabdoviridae*** |  |  |  |  |  |
|  | *Rhabdoviridae* | *Alpharicinrhavirus* | Tahe rhabdovirus 1 | NE-SL1 | ON408164 |
|  | *Rhabdoviridae* | *Betaricinrhavirus* | Yanbian rhabd tick virus 2 | TIGMIC 3 | ON746528 |
|  | *Rhabdoviridae* | *Betaricinrhavirus* | Yanbian rhabd tick virus 3 | TIGMIC 1 | ON746524 |
| ***Tombusviridae*** |  |  |  |  |  |
|  | *Tombusviridae* | *Luteovirus* | Ningxia luteovirus | China-NX143 | PP945028 |
| ***Orthototiviridae*** |  |  |  |  |  |
|  | *Orthototiviridae* |  | Daqing totiv tick virus 1 | TIGMIC_3 | ON812600 |
|  | *Orthototiviridae* |  | Toti-like virus 4 | China-NX145 | PP945342 |
|  | *Orthototiviridae* |  | Yanbian totiv tick virus 1 | TIGMIC_74 | ON812753 |
| **Unclassified** |  |  |  |  |  |
|  | Unclassified |  | Yanbian reovi tick virus 4 | TIGMIC 1 | ON746512 |

Table S3. Identified viral strains in the present study.

| **Viruses** | **Segments/genome** | **Collection sites** | **Host speciese** | **Viral strain** | **GenBank accession Nos.** |
| --- | --- | --- | --- | --- | --- |
| Tick-borne encephalitis virus | Nearly complete genome | China: Inner Mongolia, Hulunbuir | *Haemaphysalis concinna* | YKS17 | PZ672155 |
| Ji'an nariovirus | L | China: Jilin,Yanbian | *Haemaphysalis concinna* | WQ6 | PZ671672 |
| Ji'an nariovirus | M | China: Jilin,Yanbian | *Haemaphysalis concinna* | WQ6 | PZ672159 |
| Ji'an nariovirus | S | China: Jilin,Yanbian | *Haemaphysalis concinna* | WQ6 | PZ672162 |
| Songling virus | L | China: Jilin,Yanbian | *Haemaphysalis concinna* | LJ9 | PZ671673 |
| Songling virus | M | China: Jilin,Yanbian | *Haemaphysalis concinna* | LJ9 | PZ672160 |
| Songling virus | S | China: Jilin,Yanbian | *Haemaphysalis concinna* | LJ9 | PZ672163 |
| Wetland virus | L | China: Inner Mongolia,Zhalantun | *Haemaphysalis concinna* | ZLT2 | PZ671674 |
| Wetland virus | M | China: Inner Mongolia,Zhalantun | *Haemaphysalis concinna* | ZLT2 | PZ672161 |
| Wetland virus | S | China: Inner Mongolia,Zhalantun | *Haemaphysalis concinna* | ZLT2 | PZ672164 |
| Dabieshan tick virus | L | China:Jilin, Yanbian | *Haemaphysalis concinna* | HC3 | PZ672156 |
| Dabieshan tick virus | S | China:Jilin, Yanbian | *Haemaphysalis concinna* | HC3 | PZ672157 |
| Tahe rhabdovirus 1 | Nearly complete genome | China: Jilin,Yanbian | *Haemaphysalis concinna* | DH11 | PZ723403 |
| Yanbian rhabd tick virus 2 | Nearly complete genome | China: Inner Mongolia, Hulunbuir | *Haemaphysalis concinna* | CBH4 | PZ723400 |
| Yanbian rhabd tick virus 3 | Nearly complete genome | China: Jilin, Jiaohe | *Haemaphysalis concinna* | JH4 | PZ672154 |
| Ningxia luteovirus | Nearly complete genome | China: Inner Mongolia, Hulunbuir | *Haemaphysalis concinna* | EWK11 | PZ723402 |
| Daqing totiv tick virus 1 | Nearly complete genome | China: Inner Mongolia, Hulunbuir | *Haemaphysalis concinna* | EWK1 | PZ672153 |
| Toti-like virus 4 | Nearly complete genome | China: Inner Mongolia, Hulunbuir | *Haemaphysalis concinna* | EWK10 | PZ723404 |
| Yanbian totiv tick virus 1 | Nearly complete genome | China: Jilin,Yanbian | *Haemaphysalis concinna* | HL3 | PZ723401 |
| Yanbian reovi tick virus 4 | Nearly complete genome | China: Jilin,Yanbian | *Haemaphysalis concinna* | DH6 | PZ723405 |

Table S4. Summary of tick sample collection.

| **Region** | **Location** | **No. of ticks** |
| --- | --- | --- |
| **Changbai mountains** | Hunchun | 9 |
|  | Helong | 5 |
|  | Longjing | 10 |
|  | Wangqing | 7 |
|  | Antu | 1 |
|  | Dunhua | 12 |
|  | Jiaohe | 4 |
| **Sub-total** |  | **48** |
| **Daxing'an mountains** | Arun | 6 |
|  | Yakeshi | 18 |
|  | Hailar | 4 |
|  | Ewenki | 13 |
|  | Chenbarhu | 8 |
|  | Arxan | 11 |
|  | Zhalantun | 4 |
| **Sub-total** |  | **64** |
| **Total** |  | **112** |

Table S5. Nucleotide (lower left) and amino acid (upper right) sequence identities of the TBEV E gene.

|  | **YKS17** | **NE-SL4** | **NE-TH3** | **DXAL-21** | **ANE-FE-2021** | **Zabaikalye 30-00** | | **9024** | **205** | **MDJ-02** | **MDJ-03** | **MDJ01** | **Senzhang** | **Xinjiang-01** | **Primorye-332** | **WH2012** | **Sapporo-17-Io1** | **Nanporo-18-44** | **JLCB11-08** | **Primorye-371** | **TBEV-IP-1** | **TBEV-IP-2** | **HLB-T74** | **886-84** | **Zausaev** | **Sib-XJ-X5** | **EK-328** | **Hypr** | **Himalaya-1** | **Himalaya-2** |
| --- | --- | --- | --- | --- | --- | --- | --- | --- | --- | --- | --- | --- | --- | --- | --- | --- | --- | --- | --- | --- | --- | --- | --- | --- | --- | --- | --- | --- | --- | --- |
| **YKS17** | *** | 99.4 | 99.4 | 98 | 99.4 | 99.2 | 99 | | 99 | 98.8 | 98.8 | 99.2 | 99 | 99 | 99.6 | 99.2 | 99.6 | 99.2 | 99.2 | 99.6 | 99.6 | 98.8 | 99.2 | 98.8 | 97.8 | 97.2 | 97.6 | 95.6 | 94.2 | 93.8 |
| **NE-SL4** | 97.6 | *** | 99.2 | 97.8 | 99.2 | 99 | 98.8 | | 98.8 | 98.6 | 98.6 | 99 | 98.8 | 98.8 | 99.4 | 99 | 99.4 | 99 | 99 | 99.4 | 99.4 | 98.6 | 99 | 98.6 | 97.6 | 97 | 97.4 | 95.4 | 94.4 | 94 |
| **NE-TH3** | 97.5 | 97.8 | *** | 97.8 | 99.2 | 99 | 98.8 | | 99.2 | 98.6 | 98.6 | 99 | 98.8 | 98.8 | 99.4 | 99 | 99.8 | 99 | 99 | 99.4 | 99.4 | 98.6 | 99 | 98.6 | 97.6 | 97 | 97.8 | 95.4 | 94.4 | 94 |
| **DXAL-21** | 93.1 | 93.8 | 93.5 | *** | 97.8 | 97.6 | 97.8 | | 97.8 | 97.6 | 97.6 | 98 | 97.8 | 97.8 | 98 | 98 | 98 | 98 | 98 | 98.4 | 98 | 97.2 | 97.6 | 97.2 | 96.2 | 95.6 | 96 | 94.2 | 92.9 | 92.5 |
| **ANE-FE-2021** | 93.6 | 94.1 | 93.8 | 94 | *** | 99.4 | 98.8 | | 98.8 | 99 | 99 | 99.4 | 99.2 | 99.2 | 99.4 | 99.4 | 99.4 | 99.4 | 99.4 | 99.4 | 99.8 | 98.6 | 99.4 | 98.6 | 97.6 | 97 | 97.4 | 95.6 | 94 | 93.5 |
| **Zabaikalye 30-00** | 93.3 | 93.8 | 93.4 | 93.7 | 96.9 | *** | 98.6 | | 98.6 | 98.8 | 98.8 | 99.2 | 99 | 99 | 99.2 | 99.2 | 99.2 | 99.2 | 99.2 | 99.2 | 99.6 | 98.4 | 99.6 | 98.4 | 97.4 | 96.8 | 97.2 | 95.2 | 94.2 | 93.8 |
| **9024** | 93.8 | 94.2 | 94 | 95 | 93.7 | 93.6 | *** | | 99.6 | 98.2 | 98.2 | 98.6 | 98.4 | 98.4 | 99 | 98.6 | 99 | 98.6 | 98.6 | 99 | 99 | 98.8 | 98.6 | 98.4 | 97.8 | 97.2 | 97.6 | 95.4 | 94.6 | 94.2 |
| **205** | 93.5 | 93.9 | 93.8 | 95 | 93.7 | 93.4 | 97.6 | | *** | 98.2 | 98.2 | 98.6 | 98.4 | 98.4 | 99 | 98.6 | 99.4 | 98.6 | 98.6 | 99 | 99 | 98.8 | 98.6 | 98.4 | 97.8 | 97.2 | 98 | 95.4 | 95 | 94.6 |
| **MDJ-02** | 93.2 | 93.4 | 92.9 | 93.5 | 95 | 94.6 | 93.3 | | 93.8 | *** | 99.6 | 99.2 | 99.8 | 99 | 98.8 | 99.6 | 98.8 | 99.2 | 99.2 | 99.2 | 99.2 | 98 | 98.8 | 98 | 97 | 96.4 | 96.8 | 94.8 | 93.3 | 92.9 |
| **MDJ-03** | 93.2 | 93.4 | 92.9 | 93.5 | 95 | 94.6 | 93.3 | | 93.8 | 99.9 | *** | 99.2 | 99.8 | 99 | 98.8 | 99.2 | 98.8 | 99.2 | 99.2 | 99.2 | 99.2 | 98 | 98.8 | 98 | 97 | 96.4 | 96.8 | 94.8 | 93.3 | 92.9 |
| **MDJ01** | 93 | 93.2 | 92.9 | 92.7 | 94.8 | 94.4 | 93.3 | | 93.2 | 97.7 | 97.7 | *** | 99.4 | 99.4 | 99.2 | 99.6 | 99.2 | 99.6 | 99.6 | 99.6 | 99.6 | 98.4 | 99.2 | 98.4 | 97.4 | 96.8 | 97.2 | 95.2 | 93.8 | 93.3 |
| **Senzhang** | 93.3 | 93.5 | 93 | 93.6 | 95.1 | 94.6 | 93.3 | | 93.8 | 99.9 | 99.9 | 97.8 | *** | 99.2 | 99 | 99.4 | 99 | 99.4 | 99.4 | 99.4 | 99.4 | 98.2 | 99 | 98.2 | 97.2 | 96.6 | 97 | 95 | 93.5 | 93.1 |
| **Xinjiang-01** | 93 | 93.2 | 92.9 | 92.7 | 94.8 | 94.4 | 93.3 | | 93.2 | 97.7 | 97.7 | 99.7 | 97.8 | *** | 99 | 99.4 | 99 | 99.4 | 99.4 | 99.4 | 99.4 | 98.2 | 99 | 98.2 | 97.2 | 96.6 | 97 | 95 | 93.5 | 93.1 |
| **Primorye-332** | 93.3 | 93.7 | 93.3 | 94.3 | 93.6 | 92.7 | 95.3 | | 95.2 | 92.6 | 92.6 | 92.7 | 92.7 | 92.7 | *** | 99.2 | 99.6 | 99.2 | 99.2 | 99.6 | 99.6 | 98.8 | 99.2 | 98.8 | 97.8 | 97.2 | 97.6 | 95.6 | 94.2 | 93.8 |
| **WH2012** | 93 | 93.2 | 92.9 | 92.7 | 94.8 | 94.4 | 93.3 | | 93.2 | 97.8 | 97.7 | 99.7 | 97.8 | 99.7 | 92.7 | *** | 99.2 | 99.6 | 99.6 | 99.6 | 99.6 | 98.4 | 99.2 | 98.4 | 97.4 | 96.8 | 97.2 | 95.2 | 93.8 | 93.3 |
| **Sapporo-17-Io1** | 94.2 | 94.6 | 94.6 | 96.2 | 94 | 94 | 96.2 | | 95.8 | 93.5 | 93.5 | 93.3 | 93.5 | 93.3 | 95.4 | 93.3 | *** | 99.2 | 99.2 | 99.6 | 99.6 | 98.8 | 99.2 | 98.8 | 97.8 | 97.2 | 98 | 95.6 | 94.6 | 94.2 |
| **Nanporo-18-44** | 93.3 | 93.4 | 92.9 | 93.5 | 95 | 94.4 | 93.7 | | 94 | 98.1 | 98.1 | 97.6 | 98.1 | 97.6 | 92.9 | 97.6 | 93.5 | *** | 99.6 | 99.6 | 99.6 | 98.4 | 99.2 | 98.4 | 97.4 | 96.8 | 97.2 | 95.2 | 93.8 | 93.3 |
| **JLCB11-08** | 93.7 | 93.9 | 93.4 | 93.4 | 94.9 | 94.6 | 93.7 | | 93.9 | 98.3 | 98.3 | 97.8 | 98.3 | 97.8 | 92.7 | 98 | 93.5 | 98.7 | *** | 99.6 | 99.6 | 98.8 | 99.2 | 98.4 | 97.4 | 96.8 | 97.2 | 95.2 | 94.2 | 93.8 |
| **Primorye-371** | 93.5 | 94.2 | 93.9 | 97.2 | 94.3 | 94.2 | 95.5 | | 95.2 | 94 | 94 | 93.3 | 94.1 | 93.3 | 94.6 | 93.3 | 96.7 | 93.6 | 93.9 | *** | 99.6 | 98.8 | 99.2 | 98.8 | 97.8 | 97.2 | 97.6 | 95.6 | 94.2 | 93.8 |
| **TBEV-IP-1** | 93.7 | 94.1 | 93.6 | 94 | 97.5 | 98.3 | 94 | | 93.9 | 95.1 | 95.1 | 94.9 | 95.2 | 94.9 | 93.4 | 94.9 | 94.4 | 94.8 | 95 | 94.4 | *** | 98.8 | 99.6 | 98.8 | 97.8 | 97.2 | 97.6 | 95.6 | 94.2 | 93.8 |
| **TBEV-IP-2** | 88 | 88.5 | 87.9 | 87.6 | 87.8 | 87.1 | 87.6 | | 87.2 | 87.3 | 87.3 | 87.2 | 87.4 | 87.2 | 87.1 | 87.2 | 87.8 | 87.7 | 87.6 | 88.2 | 87.6 | *** | 98.4 | 98.2 | 98 | 97 | 97.4 | 95.4 | 94.6 | 94.2 |
| **HLB-T74** | 93.3 | 93.8 | 93.4 | 93.8 | 96.8 | 98.8 | 93.4 | | 93.6 | 94.7 | 94.7 | 94.7 | 94.8 | 94.7 | 92.7 | 94.7 | 93.8 | 94.5 | 94.7 | 94.2 | 98.1 | 87.2 | *** | 98.4 | 97.4 | 97.2 | 97.2 | 95.2 | 94.2 | 93.8 |
| **886-84** | 87 | 87.2 | 86.8 | 87.2 | 87.8 | 87.3 | 87.6 | | 87.6 | 86.5 | 86.5 | 86.8 | 86.6 | 86.8 | 86.7 | 86.9 | 87.9 | 87 | 86.8 | 87.2 | 87.4 | 87.2 | 87.2 | *** | 98.2 | 97.6 | 98 | 95.8 | 94.4 | 94 |
| **Zausaev** | 85.3 | 85.7 | 85.6 | 84.9 | 85.3 | 85 | 85.5 | | 84.9 | 84.9 | 84.9 | 85.2 | 85 | 85.2 | 85.3 | 85.2 | 85.6 | 85.4 | 85.3 | 85.3 | 85.3 | 85.9 | 84.9 | 87.2 | *** | 99 | 99 | 96.4 | 94 | 93.5 |
| **Sib-XJ-X5** | 85.2 | 85.6 | 85.5 | 84.7 | 85.4 | 85.1 | 85.1 | | 84.8 | 85.3 | 85.3 | 85.7 | 85.4 | 85.7 | 84.9 | 85.7 | 85.6 | 85.5 | 85.7 | 85 | 85.1 | 86.6 | 85.3 | 86.2 | 93 | *** | 98.4 | 95.6 | 93.8 | 93.3 |
| **EK-328** | 85.1 | 85.6 | 85.6 | 84.9 | 84.9 | 84.8 | 85.3 | | 85.1 | 84.8 | 84.8 | 85 | 84.9 | 84.9 | 84.3 | 84.9 | 85.8 | 85.1 | 85.1 | 85.2 | 84.9 | 85.8 | 84.7 | 87.4 | 92.5 | 92.7 | *** | 96 | 94.2 | 93.8 |
| **Hypr** | 83.5 | 83.5 | 83.7 | 83.5 | 84.5 | 83.2 | 84.1 | | 83.6 | 83.1 | 83.1 | 83.4 | 83.1 | 83.4 | 83.9 | 83.5 | 84.3 | 83.5 | 83.7 | 84.5 | 83.9 | 83.4 | 83.2 | 84 | 84.7 | 85.1 | 84.5 | *** | 92.9 | 92.7 |
| **Himalaya-1** | 83.9 | 84.3 | 84.4 | 84.1 | 84.2 | 84.5 | 84.5 | | 84.3 | 84.3 | 84.3 | 83.9 | 84.3 | 83.9 | 83.9 | 83.9 | 84.4 | 83.8 | 84 | 84.5 | 84 | 84.3 | 83.9 | 84.7 | 83.5 | 83.7 | 84.6 | 83 | *** | 99.6 |
| **Himalaya-2** | 83.6 | 84 | 84.1 | 84 | 83.9 | 84.2 | 84.3 | | 84.3 | 84.1 | 84.1 | 83.8 | 84.2 | 83.8 | 83.8 | 83.8 | 84.3 | 83.7 | 83.9 | 84.4 | 83.7 | 84 | 83.7 | 84.5 | 83.3 | 83.6 | 84.3 | 82.8 | 99.6 | *** |

*Abbreviations: TBEV, Tick-borne encephalitis virus.

|  | **DBSTV HC3** | **DBSTV TIGMIC 38** | **DBSTV HCSQ34** | **DBSTV HCSQ69** | **DBSTV HCCJ14** | **DBSTV NE-TMXP5** | **DBSTV HCCJ20** | **DBSTV HCSQ62** | **DBSTV HCCJ2** | **DBSTV TMSQ5** | **DBSTV TMSQ8** | **DBSTV JLYB-2024-1** | **DBSTV NE-BXXP1** | **DBSTV tick/Shandong/SDLY14/2019** | **YJTV YJ1-1** | **HPTV2 H114-17** | **OkTV 17ISK-T8** | **RUKV LEIV-6269C** |
| --- | --- | --- | --- | --- | --- | --- | --- | --- | --- | --- | --- | --- | --- | --- | --- | --- | --- | --- |
| **DBSTV HC3** | *** | 99.8 | 99.5 | 99.5 | 99.6 | 99.2 | 99.7 | 99.8 | 99.6 | 99.6 | 99.6 | 99.2 | 99.3 | 99.3 | 65.6 | 36.3 | 66.6 | 39 |
| **DBSTV TIGMIC 38** | 99.1 | *** | 99.6 | 99.6 | 99.7 | 99.2 | 99.8 | 99.8 | 99.7 | 99.7 | 99.6 | 99.4 | 99.4 | 99.4 | 65.6 | 36.3 | 66.5 | 39 |
| **DBSTV HCSQ34** | 98.4 | 98.9 | *** | 100 | 99.9 | 98.9 | 99.5 | 99.7 | 99.8 | 99.9 | 99.7 | 99.3 | 99.6 | 99.7 | 65.5 | 36.3 | 66.5 | 39 |
| **DBSTV HCSQ69** | 98.4 | 98.9 | 99.8 | *** | 99.9 | 98.9 | 99.5 | 99.7 | 99.8 | 99.9 | 99.7 | 99.3 | 99.6 | 99.7 | 65.5 | 36.3 | 66.5 | 39 |
| **DBSTV HCCJ14** | 98.4 | 98.8 | 99.7 | 99.7 | *** | 99 | 99.6 | 99.8 | 99.8 | 100 | 99.8 | 99.3 | 99.6 | 99.7 | 65.6 | 36.3 | 66.5 | 39 |
| **DBSTV NE-TMXP5** | 99 | 98.8 | 98.1 | 98.1 | 98.1 | *** | 99.2 | 99.2 | 99 | 99 | 98.9 | 98.6 | 98.8 | 98.8 | 65.2 | 36.1 | 66.2 | 38.8 |
| **DBSTV HCCJ20** | 98.8 | 98.9 | 98.8 | 98.8 | 98.9 | 98.6 | *** | 99.9 | 99.6 | 99.6 | 99.5 | 99.2 | 99.4 | 99.4 | 65.6 | 36.3 | 66.6 | 38.9 |
| **DBSTV HCSQ62** | 98.6 | 98.9 | 99.4 | 99.4 | 99.4 | 98.3 | 99.2 | *** | 99.7 | 99.8 | 99.7 | 99.3 | 99.5 | 99.5 | 65.7 | 36.3 | 66.6 | 39 |
| **DBSTV HCCJ2** | 98.5 | 98.9 | 99.5 | 99.4 | 99.4 | 98.3 | 98.8 | 99.3 | *** | 99.8 | 99.6 | 99.4 | 99.5 | 99.6 | 65.6 | 36.3 | 66.4 | 39 |
| **DBSTV TMSQ5** | 98.4 | 98.9 | 99.7 | 99.8 | 99.7 | 98.1 | 98.8 | 99.4 | 99.4 | *** | 99.8 | 99.3 | 99.6 | 99.7 | 65.6 | 36.3 | 66.5 | 39 |
| **DBSTV TMSQ8** | 98.2 | 98.7 | 99.5 | 99.6 | 99.4 | 97.9 | 98.6 | 99.2 | 99.2 | 99.6 | *** | 99.2 | 99.5 | 99.5 | 65.6 | 36.4 | 66.6 | 39 |
| **DBSTV JLYB-2024-1** | 98.4 | 98.5 | 98.8 | 98.8 | 98.7 | 98.1 | 98.7 | 98.8 | 98.7 | 98.8 | 98.7 | *** | 99.1 | 99.2 | 65.3 | 36.2 | 66.2 | 38.9 |
| **DBSTV NE-BXXP1** | 98 | 98.4 | 99.3 | 99.2 | 99.1 | 97.7 | 98.3 | 98.9 | 99 | 99.3 | 99.1 | 98.4 | *** | 99.4 | 65.3 | 36.2 | 66.3 | 39 |
| **DBSTV tick/Shandong/SDLY14/2019** | 98.1 | 98.5 | 99.3 | 99.4 | 99.3 | 97.8 | 98.5 | 98.9 | 99.1 | 99.4 | 99.2 | 98.7 | 98.9 | *** | 65.6 | 36.3 | 66.4 | 39.1 |
| **YJTV YJ1-1** | 65 | 65.1 | 65 | 65.1 | 65.1 | 64.9 | 65 | 65.1 | 65 | 65.1 | 65 | 65 | 65 | 65.1 | *** | 36.9 | 76.7 | 39.2 |
| **HPTV2 H114-17** | 46.1 | 46.2 | 46.2 | 46.2 | 46.1 | 46 | 46.2 | 46.2 | 46.1 | 46.2 | 46.3 | 46.2 | 46 | 46.1 | 47.3 | *** | 36.6 | 49.2 |
| **OkTV 17ISK-T8** | 65 | 64.9 | 64.9 | 64.9 | 64.9 | 64.8 | 64.9 | 65.1 | 64.9 | 65 | 65 | 64.9 | 64.8 | 65 | 70.8 | 46.9 | *** | 39 |
| **RUKV LEIV-6269C** | 46 | 46.1 | 46.2 | 46.2 | 46.2 | 46 | 46.1 | 46.1 | 46.1 | 46.1 | 46.2 | 46.1 | 46.1 | 46.2 | 46.2 | 55.2 | 46.7 | *** |

Table S6. Nucleotide (lower left) and amino acid (upper right) sequence identities of the DBSTV RdRp gene.

Abbreviations: DBSTV, Dabieshan tick virus; YJTV, Yongjia Tick Virus; HPTV2, Huangpi Tick Virus 2; OkTV, Okutama tick virus; RUKV, Rukutama virus.

Table S7. Nucleotide (lower left) and amino acid (upper right) sequence identities of SGLV, JANV and WELV RdRp gene.

|  | **SGLV**  **CBH6** | **SGLV NE-GP** | **SGLV NE-GH** | **SGLV NE-WE** | **JANV**  **WQ6** | **JANV NE-SDG2** | **ATV YB tick 2021 24** | **YBNTV2 TIGMIC 1** | **WELV**  **ZLT2** | **WELV T3G2** | **WELV PS1** | **WELV T8A1** | **YBNTV1** | **SXTV2 NE-HC5** | **NSDV 708** | **TcTV-1 QH1** |
| --- | --- | --- | --- | --- | --- | --- | --- | --- | --- | --- | --- | --- | --- | --- | --- | --- |
| **SGLV CBH6** | *** | 99.6 | 99.7 | 99.6 | 85.3 | 85.3 | 85.2 | 85.2 | 38.5 | 38.4 | 38.4 | 38.4 | 81 | 66.9 | 36.9 | 64.5 |
| **SGLV NE-GP** | 98.7 | *** | 99.6 | 99.5 | 85.3 | 85.3 | 85.2 | 85.3 | 38.5 | 38.5 | 38.5 | 38.5 | 81 | 66.9 | 36.9 | 64.6 |
| **SGLV NE-GH** | 98.6 | 98.6 | *** | 99.7 | 85.3 | 85.3 | 85.2 | 85.2 | 38.5 | 38.4 | 38.4 | 38.4 | 81 | 66.9 | 36.9 | 64.6 |
| **SGLV NE-WE** | 97.5 | 97.4 | 97.4 | *** | 85.5 | 85.5 | 85.4 | 85.4 | 38.5 | 38.4 | 38.4 | 38.4 | 81 | 66.9 | 36.8 | 64.5 |
| **JANV WQ6** | 73.8 | 73.8 | 73.8 | 74 | *** | 100 | 99.8 | 99.9 | 38.1 | 38.1 | 38.1 | 38.1 | 77.9 | 66.8 | 37 | 64.2 |
| **JANV NE-SDG2** | 73.7 | 73.6 | 73.7 | 73.9 | 99.4 | *** | 99.8 | 99.9 | 38.1 | 38.1 | 38.1 | 38.1 | 78 | 66.8 | 37 | 64.2 |
| **ATV YB tick 2021 24** | 73.7 | 73.6 | 73.7 | 73.9 | 99.2 | 99.3 | *** | 99.8 | 38.1 | 38.1 | 38.1 | 38.1 | 78 | 66.8 | 37 | 64.3 |
| **YBNTV2 TIGMIC 1** | 73.7 | 73.6 | 73.7 | 73.9 | 99.4 | 99.4 | 99.7 | *** | 38.1 | 38.1 | 38.1 | 38.1 | 77.9 | 66.8 | 37 | 64.2 |
| **WELV ZLT2** | 47.4 | 47.4 | 47.4 | 47.4 | 46.9 | 46.9 | 46.9 | 46.9 | *** | 99.9 | 99.8 | 99.9 | 38.2 | 38.2 | 68.8 | 38.6 |
| **WELV T3G2** | 47.4 | 47.4 | 47.4 | 47.4 | 46.9 | 46.8 | 46.9 | 46.9 | 99.8 | *** | 99.9 | 100 | 38.2 | 38.2 | 68.8 | 38.6 |
| **WELV PS1** | 47.4 | 47.4 | 47.4 | 47.4 | 46.9 | 46.8 | 46.9 | 46.9 | 99.8 | 100 | *** | 99.9 | 38.2 | 38.2 | 68.8 | 38.6 |
| **WELV T8A1** | 47.4 | 47.4 | 47.4 | 47.5 | 46.9 | 46.9 | 46.9 | 46.9 | 99.8 | 99.9 | 99.9 | *** | 38.2 | 38.2 | 68.8 | 38.6 |
| **YBNTV1** | 74.6 | 74.5 | 74.7 | 74.3 | 69.5 | 69.5 | 69.5 | 69.5 | 47.4 | 47.4 | 47.4 | 47.4 | *** | 68.2 | 36.8 | 65.8 |
| **SXTV2 NE-HC5** | 63.9 | 64 | 63.9 | 63.9 | 63.8 | 63.8 | 63.7 | 63.8 | 47 | 47 | 47 | 47 | 64.2 | *** | 37.5 | 64.4 |
| **NSDV 708** | 46.3 | 46.2 | 46.3 | 46.2 | 46.2 | 46.2 | 46.2 | 46.2 | 64.7 | 64.7 | 64.7 | 64.7 | 45.9 | 46.4 | *** | 37.5 |
| **TcTV-1 QH1** | 62.6 | 62.5 | 62.5 | 62.5 | 62.2 | 62 | 62.1 | 62.1 | 47.8 | 47.8 | 47.8 | 47.9 | 63.2 | 62.8 | 46.8 | *** |

Abbreviations: SGLV, Songling virus; JANV, Ji'an nariovirus; WELV, Wetland virus; ATV, Antu virus; YBNTV2, Yanbian nairo tick virus 2; YBNTV1, Yanbian nairo tick virus 1; SXTV2, Shanxi tick virus 2; NSDV, Nairobi sheep disease virus; TcTV-1, Tacheng Tick

Figure S1. Composition and relative abundance of viral families identified in the samples.

Figure S2. Standard curves for the 11 tick-borne viruses. To establish standard curves, 11 tick-borne virus gene fragments were separately cloned into the pMD19-T vector to construct the required plasmids. The results showed the regression equations and determination coefficient (R²) as follows: Y = -3.33X + 40.29 (R²: 1) for Wetland virus (WELV); Y = -3.53X + 43.38 (R²: 0.998) for Ji’an nairovirus (JANV); Y = -3.60X + 42.20 (R²: 0.996) for Tahe rhabdovirus 1 (THRV1); Y = -3.51X + 42.84 (R²: 0.999) for Dabieshan tick virus (DBSTV); Y = -3.48X + 42.18 (R²: 0.999) for Yanbian rhabd tick virus 2 (YBRV2); Y = -3.46X + 45.44 (R²: 0.999) for Daqing totiv tick virus 1 (DQTV1), Y = -3.64X + 43.13 (R²: 0.998) for Ningxia luteovirus (NXLV); Y = -3.51X + 42.85 (R²: 1) for YBRTV4; Y = -3.69X + 43.47 (R²: 0.998) for Yanbian rhabd tick virus 3 (YBRV3); Y = -3.83X + 46.10 (R²: 0.999) for Yanbian totiv tick virus 1 (YBTV1); and Y = -3.25X + 38.24 (R²: 0.998) for Toti-like virus 4 (TLV4).
